# Clinical analysis of secondary hemophagocytic syndrome in children in Gansu province

**DOI:** 10.3389/fped.2025.1660880

**Published:** 2025-11-06

**Authors:** Yongkang Zhou, Junqiang Yang, Zhongbin Tao

**Affiliations:** The Pediatrics of The First Hospital of Lanzhou University, The First Clinical Medical College of Lanzhou University, Lanzhou, China

**Keywords:** visceral leishmaniasis, secondary, hemophagocytic lymphohistiocytosis, Epstein–Barr virus, children

## Abstract

**Objective:**

This study aims to explore and analyze the various causes, clinical features, and prognostic differences of secondary hemophagocytic syndrome (SHS) in children in Gansu Province, China.

**Method:**

This study used a retrospective analysis method, selected 43 children with HLH who were treated in the PICU at the First Hospital of Lanzhou University from January 2019 to January 2025. Based on their primary diseases, the subjects were categorized into groups for VL, EB virus infection, and other infections. The clinical manifestations, laboratory results and clinical outcomes of each group were comprehensively analyzed.

**Results:**

The main cause of HLH was infection (35/43 cases), with EB virus infection (15/35 cases) and leishmania (13/35 cases) being the most common. In the leishmania and EB virus infection group, the average of alanine aminotransferase (ALT) levels were 81 U/L and 87 U/L respectively, were higher than other infection group (*P* < *0.05*). The average of procalcitonin (PCT) levels were 2.88 ng/mL, 4.37 ng/mL respectively in the Leishmania group and other infection group, were higher than EBV infection group(0.43 ng/mL). The average of D-dimer (D-D) levels were statistically significant differences among Leishmania group(2.31 mg/dL), EBV infection group(2.12 mg/dL) and the other infection group (3.34 ng/mL). The leishmania group had a cure rate of 100% (13/13), while the EB virus infection group had a cure rate of 26.67% (4/15).

**Conclusion:**

Leishmania and EB virus infection are common causes of HLH in Gansu Province. Compared to HLH caused by other factors such as EB virus infection or autoimmune diseases, the prognosis of HLH in children with leishmania infection is relatively better.

## 1 Introduction

Hemophagocytic syndrome (HPS), also known as hemophagocytic lymphohistiocytosis (HLH), is a rare and life-threatening condition characterized by excessive inflammatory response. This syndrome features abnormal activation of the immune system, a cytokine storm effect, and aberrant proliferation of histiocytes. The core pathological mechanisms involve dysregulation of immune cells, including T cells, natural killer cells, and macrophages, leading to the release of large amounts of pro-inflammatory cytokines such as IL-6, IFN-γ, and TNF-α, which result in multi-organ damage. Hemophagocytic syndrome can be classified into two major categories based on etiology: primary (genetic) and secondary (acquired) (1).

Secondary hemophagocytic lymphohistiocytosis (sHLH) is a syndrome characterized by excessive inflammatory response triggered by clear inducements such as infections, tumors, and autoimmune diseases, accounting for the majority of hemophagocytic lymphohistiocytosis (HLH) cases. Unlike primary HLH (genetic), secondary HLH does not have identifiable gene mutations. Among infectious causes, Epstein–Barr virus (EBV) infection is predominant (2). However, in the Gansu region of China, due to its location at the junction of the Loess Plateau and the Qinghai-Tibet Plateau, where livestock farming is well-developed, hemophagocytic syndrome caused by local diseases such as kala-azar and hemorrhagic fever is relatively common, especially prominent in the Longnan area of Gansu (3, 4). Kala-azar, also known as visceral leishmaniasis (VL), is a chronic parasitic disease caused by infection with Leishmania donovani, transmitted through the bite of sandflies. The disease primarily affects visceral organs such as the liver, spleen, and bone marrow, characterized by prolonged fever, hepatosplenomegaly, pancytopenia, and hyperimmunoglobulinemia. It often leads to secondary hemophagocytic syndrome, with a mortality rate exceeding 90% if left untreated (5).

## 2 Method

### 2.1 Case collection

We performed genetic testing on all cases and selected cases without HLH-related gene mutations for inclusion in the study, Total collected case data of 43 children with secondary hemophagocytic lymphohistiocytosis admitted to the Pediatric Intensive Care Unit of the First Hospital of Lanzhou University from January 2019 to June 2025.

### 2.2 Diagnostic criteria

The Chinese Guidelines for the Diagnosis and Treatment of Hemophagocytic Lymphohistiocytosis (2022 Edition) recommend that the diagnostic and classification criteria for HLH be based on the HLH-2004 diagnostic criteria (6). The diagnosis of MAS refers to the 2016 edition criteria (7). Other factors such as HLH inflammation index (8), flow cytometry testing, and cytokines (9) were not included in the diagnostic criteria due to objective limitations. The diagnostic criteria for kala-azar were formulated according to the Law of the People's Republic of China on the Prevention and Treatment of Infectious Diseases and its Implementation Measures. The confirmed diagnosis requires a history of residence in an endemic area along with clinical symptoms, and the presence of Leishman bodies in the bone marrow; for clinically diagnosed cases, at least one of the immunological tests including direct agglutination test, indirect fluorescent antibody test, rk39 immunochromatographic strip method, or enzyme-linked immunosorbent assay must be positive.

### 2.3 Treatment methods

Treatment of the primary disease: The Leishmania infection group were treated with sodium antimony gluconate, the EBV group were treated with acyclovir; the other infections were treated with antibiotics. Immunosuppressants were used in MAS cases, and malignancy were treated with standardized chemotherapy regimens. The Leishmania infection group first received treatment with stibogluconate for 5 days, and those without improvement in clinical symptoms initiated the HLH treatment protocol.

HLH Treatment Protocol: First we used 8-week induction treatment with etoposide (VP-16), and dexamethasone, then treated with the DEP salvage regimen (Doxorubicin, Etoposide, and Methylprednisolone) which did not achieve partial response (PR) or higher efficacy.

Due to objective conditions in the Gansu region, salvage treatment regimens for HLH such as targeted drugs (10), immunotherapy (11), and gene therapy were not routinely adopted.

### 2.4 Analysis method

This study conducted a retrospective analysis of the etiology, clinical manifestations, laboratory tests, diagnosis and treatment, and outcomes of 43 children with secondary hemophagocytic lymphohistiocytosis (HLH) admitted to the Pediatric Intensive Care Unit (PICU) of the First Hospital of Lanzhou University from January 2019 to June 2025.

### 2.5 Statistical analysis

This study used SPSS version 22.0 software for statistical analysis. Measurement data were expressed as $\bar{X}\pm s$, and intergroup comparisons were performed using one-way ANOVA; count data were expressed as percentages, and chi-square tests were used. *P* *<* *0.05* indicated statistically significant differences.

## 3 Results

This study conducted a statistical analysis of the clinical data of 43 cases of secondary hemophagocytic lymphohistiocytosis (HLH) patients. The clinical diagnostic criteria included fever, splenomegaly, cytopenia etc (Table 1). The primary cause of secondary HLH is infection, while secondary causes include autoimmune diseases and malignancies.

**Table 1:** Clinical manifestations of secondary HLH.

Secondary factor	Fever	Splenomegaly	Cytopenia	TG	FIB	Hemophagocytosis	NK cells	ferropr-otein	sCD25
MAS (5)	5	5	5	4	4	4	5	5	4
Malignant tumor (3)	3	3	3	2	1	3	3	3	2
Infection (35)	35	35	35	16	17	23	31	32	30

TG, Triglyceride; FIB, Fibrinogen; NK cell, Natural Killer Cells; sCD25, Soluble Interleukin-2 Receptor; MAS, Macrophage Activation Syndrome.

The analysis of alanine aminotransferase (ALT) levels in secondary HLH cases showed that the highest ALT level in the Leishmania infection group was 81 U/L, with an average of 50.69 U/L. Seven children had ALT levels ≥50 U/L. In the EB virus infection group, the highest ALT level was 87 U/L, with an average of 49.87 U/L. Six children had AST levels ≥50 U/L. There was no significant increase in ALT levels in other infection group. The differences between the groups were statistically significant (Table 2). Multiple comparisons revealed that there were statistically significant differences in ALT levels between the Leishmania infection group, or EB virus infection group, and other infection group (*P* < *0.05)*.

**Table 2:** One-factor ANOVA of ALT in patients with infection-related HLH.

Pathogen	*N*	$\bar{X}$	SD	Min	Max	*F*	*P*
Leishmania	13	50.69	17.289	24	81	*5.168*	*0.011*
EBV	15	49.87	25.796	19	87		
Others	7	22.71	6.55	13	33		
Total	35	44.74	22.629	13	87		

*N*, Number of cases; $\bar{X}$, Mean; SD, Standard deviation; Min, Minimum value; Max, Maximum value; *F*, *F*-statistic value; *P*, *P*-value (*P* < 0.05 indicates statistical significance).

The analysis of procalcitonin (PCT) levels showed that in the Leishmania infection group, PCT levels reached a peak of 11 ng/mL, with an average of 2.88 ng/mL, and PCT ≥ 0.5 ng/mL was observed in 9 children; in the EB virus infection group, PCT levels peaked at 1.01 ng/mL, with an average of 0.43 ng/mL, and PCT ≥ 0.5 ng/mL was observed in 5 children; in the other infection group, PCT levels increased more significantly, reaching a peak of 11.4 ng/mL, with an average of 4.37 ng/mL. The differences among the three groups were statistically significant (Table 3).

**Table 3:** One-factor ANOVA of PCT in patients with infection-related HLH.

Pathogen	*N*	$\bar{X}$	SD	Min	Max	*F*	*P*
Leishmania	13	2.88	3.75	0.25	11	*5.701*	*0.008*
EBV	15	0.43	0.26	0.18	1.01		
Others	7	4.37	3.41	0.98	11.4		
Total	35	2.13	3.09	0.18	11.4		

*N*, Number of cases; $\bar{X}$, Mean; SD, Standard deviation; Min, Minimum value; Max, Maximum value; *F*, *F*-statistic value; *P*, *P*-value (*P* < 0.05 indicates statistical significance).

The analysis of D-dimer (D-D) levels revealed that in the Leishmania infection group, the highest D-D level was 11 mg/dL, with an average of 2.31 mg/dL, and 9 children had a D-D level of ≥1 mg/dL. In the EB virus infection group, the highest D-D level was 2.12 mg/dL, with an average of 0.69 mg/dL, and 3 children had a D-D level of ≥1 mg/dL. The other infection group showed more significant increases in D-D levels, with the highest being 6.21 ng/mL, and an average of 3.34 ng/mL. The differences among the three groups were statistically significant (Table 4). Multiple comparisons indicated that there were statistically significant differences in D-D levels between the Leishmania infection group, the other infection group, and the EB virus infection group (*P* < *0.05*).

**Table 4:** One-factor ANOVA of D-D in patients with infection-related HLH.

Pathogen	*N*	$\bar{X}$	SD	Min	Max	*F*	*P*
Leishmania	13	2.31	1.38	0.16	4.3	*14.108*	<*0.05*
EBV	15	0.69	0.51	0.21	2.12		
Others	7	3.34	1.68	1.63	6.21		
Total	35	1.82	1.55	0.16	6.21		

*N*, Number of cases; $\bar{X}$, Mean; SD, Standard deviation; Min, Minimum value; Max, Maximum value; *F*, *F*-statistic value; *P*, *P*-value (*P* < 0.05 indicates statistical significance).

**Figure 1:**
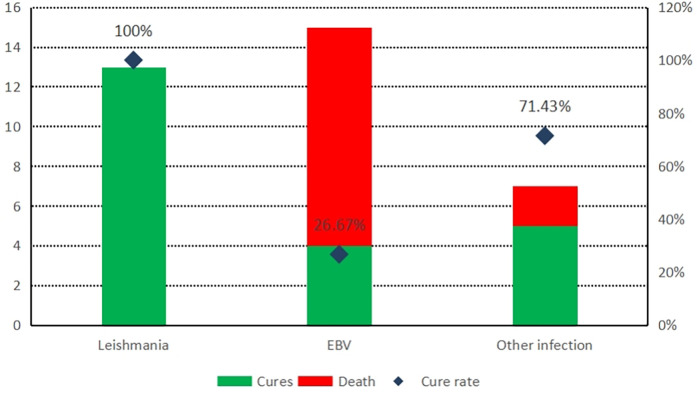
Comparison of prognosis of HLH with different infections.

In all cases of infection-related HLH, the leishmania infection group showed the best prognosis with no death; in contrast, the EBV infection group had a cure rate of only 26.67% (Figure 1), and the difference between the two groups was statistically significant (Table 5). However, there was no statistically significant difference in cure rates between other groups (MAS, malignant tumors, and other infections) and the EBV group or the HLH group.

**Table 5:** Analysis of HLH outcomes associated with infection.

Pathogen	Cure rate	Death	*χ^2^*	*P*	*OR*
Leishmania	13	0	*15.702*	*<0.01*	*3.75*
EBV	4	11			

*χ²*, Chi-square value; *P*, *P*-value (*P* < 0.05 indicates statistical significance); *OR,* Odds ratio*.*

## 4 Discussion

HLH is a syndrome characterized by excessive inflammation and tissue damage due to abnormal immune activation. The underlying pathophysiology involves dysregulation of the body's immune system, leading to impaired downregulation of macrophages and lymphocytes. In most patients with HLH, the cytotoxic functions of NK cells and cytotoxic T lymphocytes (CTLs) are compromised, accompanied by excessive activation of macrophages. Clinically, it is marked by persistent fever, hepatosplenomegaly, pancytopenia, and the presence of hemophagocytosis in bone marrow, liver, spleen, and lymphoid tissues. Secondary (sporadic or acquired) HLH refers to cases without family mutations, often triggered by specific acute conditions such as infections, tumors, and rheumatic diseases. Among these, viral infections, particularly EB virus, are the primary cause of infection-related HLH, while infections from other pathogens like bacteria, parasites, and fungi are relatively rare (2). In this study, secondary hemophagocytic syndrome in children was mainly caused by EB virus infection and Leishmania infection, which was different from the epidemiological characteristics of other places at domestic areas and abroad. It was mainly related to many factors such as large area and sparse population, large number of agricultural and animal husbandry population and relatively backward medical and health conditions in Gansu province.

Kala-azar is endemic in many tropical regions and the Mediterranean basin around the world (12), it is endemic in Xinjiang, Gansu, Sichuan, Shaanxi and Shanxi provinces. Kala-azar has a long incubation period, ranging from 2 weeks to 18 months. Children typically develop symptoms within 2–8 months after infection, especially if bitten by sandflies during the peak season (June to July), with most cases occurring in winter and spring. The main clinical manifestations of visceral leishmaniasis (VL) include irregular fever, pancytopenia, and hepatosplenomegaly. This condition may be due to the Leishmania parasite multiplying extensively in the reticuloendothelial system after invading the host, thereby evading both innate and cellular-mediated immune responses, leading to excessive activation of macrophages and the production of large amounts of cytokines (13). In this study, children with kala-azar exhibited significant splenomegaly, and half of the patients also had elevated liver enzymes. This is due to the parasites proliferating in the reticuloendothelial system, leading to a substantial accumulation of parasites in the spleen, liver, and bone marrow. Studies abroad have shown that there is a certain rate of false negatives in diagnosing kala-azar through serological tests especially in immunosuppressed or immunocompromised patients, so it is recommended to use PCR testing to enhance diagnostic sensitivity (12). Due to the limitations of objective conditions, this study confirmed the presence of Leishmania ameboides through bone marrow smear and rk39 antibody test. When no Leishmania ameboides was found in the bone marrow smear, the rk39 antibody test still showed high sensitivity (13), which can reduce missed or misdiagnoses.

In the world, such as Europe, North America and South America, amphotericin B is recommended as the first choice for treating kala-azar (14, 15). However, the high cost of liposomal amphotericin B limits its use in impoverished areas. Considering Chinese national conditions, the 2017 edition of the’ Expert Consensus on Diagnosis and Treatment of Leishmaniasis Infection in China’ recommends that due to its low cost, easy availability, and extensive application experience, antimony agents remain the preferred treatment option. In this study, all cases of kala-azar were treated with sodium antimony gluconate. After standardized treatment for the primary disease, the clinical manifestations related to HLH rapidly improved, and never initiated VP-16 chemotherapy regimen, resulting in a generally favorable prognosis.

Epstein–Barr virus (EBV) belongs to the herpesvirus family and is one of the pathogens that cause long-term human infection (16), about 90 percent of the global population is infected (17). Most EBV infections are subclinical or occult. Clinically, the most common manifestation of EBV infection in children is infectious mononucleosis (IM), characterized by fever, pharyngitis, and enlarged cervical lymph nodes, often accompanied by symptoms such as hepatosplenomegaly and eyelid swelling. IM is typically a self-limiting condition, with a good prognosis in most cases. However, EBV infection can also lead to serious conditions, such as HLH or malignant tumors (18). Among all the factors leading to HLH in children, EBV infection is the most common cause. According to the statistical analysis of 2019 HLH cases in China (19), Among the 1,445 cases across 31 regions, EBV infection was the leading cause, accounting for 44.01%. In this study, EBV infection was one of the primary causes. During the follow-up period, 11 patients in the EBV-HLH group unfortunately passed away during treatment, while only 4 patients survived and continued to be followed up. This finding reaffirms the poor clinical prognosis of EBV-HLH cases.

This study indicates that in all cases of infection-related HLH, patients with VL-HLH and EBV-HLH were more likely to experience liver function impairment. This is consistent with the tendency of both VL (20) and EB virus to affect the liver. In EBV-HLH cases, the elevation of procalcitonin (PCT) and D-dimer (D-D) levels was not significant, which aligns with the clinical features of viral infections. In contrast, in cases of Leishmania and other pathogen infections, the elevation of PCT and D-D levels was more common, especially in cases of other pathogens. This phenomenon is consistent with the elevated inflammatory markers following severe bacterial infections (21) and the hypercoagulable state resulting from vascular endothelial damage (22).

## 5 Conclusion

In the Gansu region, pediatric kala-azar continues to be prevalent. Both EB virus infection and kala-azar are common causes of HLH. Kala-azar has diverse clinical manifestations and lacks specific symptoms, making it prone to misdiagnosis and missed diagnosis. If pediatric VL-HLH is identified early and treated with antimonials, most cases have better prognosis and can avoid further VP-16 treatment. In contrast, HLH associated with EB virus infection has a higher mortality rate and a poorer prognosis.

## Data Availability

The original contributions presented in the study are included in the article/Supplementary Material, further inquiries can be directed to the corresponding author.
